# Une carotidodynie révélant une artérite de Takayasu chez un homme de 54 ans

**DOI:** 10.11604/pamj.2015.20.135.5825

**Published:** 2015-02-16

**Authors:** Faten Frikha, Zouhir Bahloul

**Affiliations:** 1 Service de Médecine Interne CHU Hédi Chaker,Tunisie

**Keywords:** artérite de Takayasu, vascularite, imagerie, Takayasu arteritis, vascularitis, imagery

## Image en medicine

L'artérite de Takayasu (AT) est une panartérite inflammatoire rare de cause inconnue, touchant les artères de gros et de moyen calibre. Elle survient essentiellement chez la femme jeune. Sa survenue chez la population masculine est rarement rapportée. Un homme âgé de 54 ans était hospitalisé pour carotidodynie gauche associée à un amaigrissement de 18 kg en six mois. L'examen clinique était sans particularités. Il avait à la biologie une anémie à 10 g/dl, un syndrome inflammatoire et un bilan lipidique correct. L'echodoppler (A) et l'angioscanner (B) des troncs supra-aortiques objectivaient un épaississement étendu et fusiforme de l'artère carotide primitive gauche avec une sténose estimée à 60% de l'artère carotide interne droite. L'enquête infectieuse, le bilan immunologique et de thrombophilie étaient négatifs. Le diagnostic d'une AT a été retenu. Le patient a reçu une corticothérapie à la dose initiale de 1 mg/kg/j. L'évolution était favorable avec disparition des carotidodynies et régression du syndrome inflammatoire biologique.

**Figure 1:**
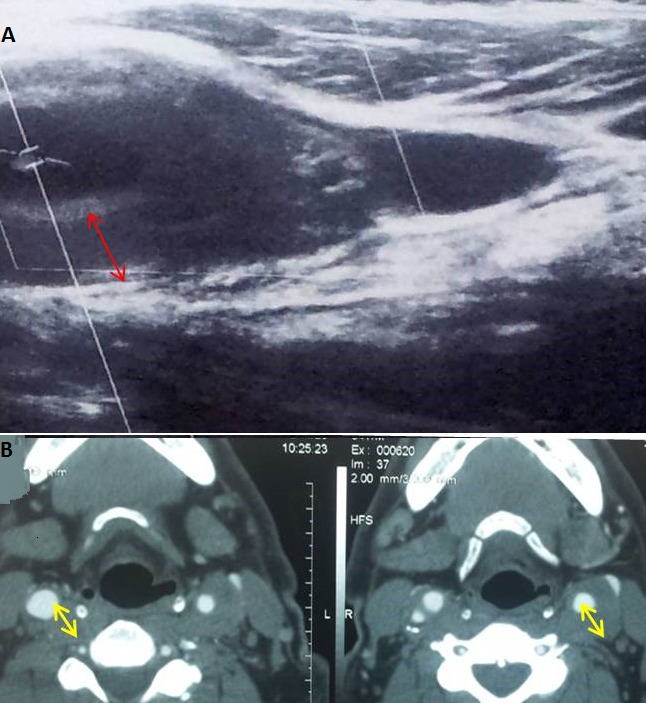
A) échographie Doppler des vaisseaux supra-aortiques montrant un épaississement de l'artère carotide commune (flèche rouge). B) angioscannar des vaisseaux supra-aortiques objectivant un épaississement étendu et fusiforme de l'artère carotide primitive gauche avec une sténose estimée à 60% de l'artère carotide interne droite (flèches jaunes)

